# Anxiety predicts internet addiction, which predicts depression among male college students: A cross-lagged comparison by sex

**DOI:** 10.3389/fpsyg.2022.1102066

**Published:** 2023-01-16

**Authors:** Xiaoqian Xie, Hui Cheng, Zi Chen

**Affiliations:** School of Psychology, Chengdu Medical College, Chengdu, China

**Keywords:** depression, anxiety, internet addiction, sex, college students

## Abstract

**Objectives:**

Internet addiction has become an increasingly serious public health issue, putting young people at particular risk of psychological harm. This study aimed to analyze the interactions between college students’ depression, anxiety, and Internet addiction and explore how these interactions differ between men and women.

**Methods:**

A 6-month follow-up study was conducted on 234 college students using the Self-Rating Depression Scale, Self-rating Anxiety Scale, and Revised Chen Internet Addiction Scale.

**Results:**

Depression, anxiety, and Internet addiction were positively correlated (*p* < 0.01). Anxiety can predict Internet addiction and that Internet addiction can predict depression. Moreover, anxiety had a significant predictive effect on Internet addiction among men.

**Conclusion:**

Anxiety predicts Internet addiction, and Internet addiction predicts depression among male college students. These findings may better inform future Internet addiction intervention strategies. Particularly, interventions may better address Internet addiction by focusing on the role of anxiety, especially among men.

## 1. Introduction

With urbanization, the Internet has become increasingly convenient, cheap, and rife with addictive content (Ko et al., 2022). Moreover, as Internet use has increased rapidly worldwide, Internet addiction has become a serious public health problem for all groups (Hassan et al., 2020). Internet addiction is an impulse control disorder in which excessive Internet use results in the neglect of real-life relationships, work, and normal daily life (Young, 1998, 2004, 2007).

Internet addiction has been found to be in co-morbidity with other psychological symptoms and psychiatric disorders (Otsuka et al., 2020), Internet addiction has been found to be associated with depression (Lau et al., 2018) and anxiety symptoms (Cai et al., 2021), insomnia (Goel et al., 2021), academic failure (Kuo et al., 2018), interpersonal withdrawal (Kato et al., 2020), and aggressive behavior (Zhao et al., 2022). Internet addiction and poor mental health status each increased the risk of onset of the other (Otsuka et al., 2020).

Currently, there is a high detection rate of internet addiction among college students (Al Shawi et al., 2021), and the impact of Internet addiction is particularly significant for college students (Shen et al., 2020), as they are still undergoing psychosocial development. Baturay and Toker, (2019) reported that Internet addiction decreases college students’ self-esteem, self-confidence, social self-efficacy, academic self-efficacy and triggers loneliness.

Compared with other maladaptive problems, Internet addiction is strongly correlated with anxiety and depression (Li et al., 2019; Andrade et al., 2020). Emotional problems, of which depression and anxiety are the most common (Tsai et al., 2020), and the comorbidity rate is high (Zeng et al., 2019), can mediate other psychological and behavioral problems (Warren et al., 2021); moreover, difficulty in emotional regulation can predict subsequent Internet addiction (Effatpanah et al., 2020). Among college students, emotional problems are more common, particularly depression and anxiety (Ramón-Arbués et al., 2020).

The influence of depression and anxiety on Internet addiction has been demonstrated in many previous studies (Christ et al., 2020; Sayed et al., 2022). Evren et al. (2019) found that the severity of Internet addiction relates to the levels of depression and anxiety. Depression is also more common among Internet addicts and over-users (Kim et al., 2016; Tan et al., 2016), and depressive symptoms have the highest predictive ability for Internet addiction (Przepiorka et al., 2019; Diotaiuti et al., 2022a). Furthermore, Karaer and Akdemir (2019) found that greater anxiety is an important predictor of Internet addiction, and Internet addiction is related to an increase in anxiety (Shen et al., 2020; Gao et al., 2021). Morita et al. (2022) conducted a three-year longitudinal study and found a two-way relationship between Internet addiction and depressive symptoms.

Internet addiction differs by sex (Liang et al., 2016). Men are more prone to Internet addiction than women (Chi et al., 2020). Researchers have identified that men showed higher levels of Internet addiction, this is related to men being more dependent, more impulsive and more interdependent (Diotaiuti et al., 2022b). A follow-up survey of 1,715 adolescents showed that depressive symptoms had a more significant predictive effect on Internet addiction among male adolescents, indicating that depression can lead to Internet addiction. Conversely, among female adolescents, Internet addiction can significantly predict subsequent depression, indicating that Internet addiction can lead to depression (Yi and Li, 2021).

Some studies have found that women with Internet addiction are more likely to have depressive symptoms (Li et al., 2019), whereas men with Internet addiction are more likely to have anxiety symptoms (Shan et al., 2021). These results show that the relationship between Internet addiction and depression varies by sex.

Many previous studies have investigated Internet addiction, but the main focus has been the bivariate study of Internet addiction and other factors rather than the relationship between depression, anxiety, and Internet addiction. Moreover, previous studies on the relationship between depression, anxiety, and Internet addiction were mostly cross-sectional; longitudinal studies have been relatively few, and there has been a lack of research on the long-term mechanism of depression, anxiety, and Internet addiction. Furthermore, to date, few studies have focused on sex-related differences in depression, anxiety, and Internet addiction. Therefore, this study adopted a longitudinal approach to explore the mutual influence and dynamic relationship between depression, anxiety, and Internet addiction in college students. In this exploration, this study aimed to clarify the relationship between Internet addiction, depression, and anxiety as well as clarify the mechanisms of Internet addiction itself.

Therefore, the purpose of this study is to explore the mutual influence and dynamic relationship between depression, anxiety, and Internet addiction in college students. Based on the findings of our literature review, we arrived at the following hypotheses: Hypothesis 1. There was a sex difference in Internet addiction. Hypothesis 2. Depression, and anxiety were positively associated with Internet addiction. Hypothesis 3. Depression and anxiety significantly predicted subsequent Internet addiction. Hypothesis 4. Internet addiction significantly predicted subsequent depression and anxiety.

## 2. Methods

### 2.1. Participants

This study used convenience sampling to select college students from a college in Sichuan Province for a follow-up study. In this study, half of the classes with psychological commissioners were randomly selected by using the psychological commissioners system of the college, and the sampling of this study was completed by issuing and retrieving questionnaires from psychological commissioners. Moreover, they were assured anonymity and provided their written informed consent to participate in this study. Students completed questionnaires at three time points: June, September, and December 2021. Data were collected in the classroom every 3 months through a paper and pencil test. Each participant had a unique ID and used the same ID in all three waves. There were 443 participants in the first wave, 281 in the second wave, and 243 in the third wave. The 243 students (baseline age 19.74 ± 0.94 years) who participated in all three waves were included in this study, and they are the data analysis objects of this study; The final sample included 90 men (baseline age 19.88 ± 1.90 years) and 153 women (baseline age 19.65 ± 0.83 years). There was no difference in the average age by sex (*t* = 1.69, *p* = 0.09). All procedures performed in this study involving human participants were in accordance with the ethical standards of the ethics committee of research institutions and with the 1964 Helsinki Declaration and its later amendments or comparable ethical standards. Additionally, participants provided informed consent.

### 2.2. Measures

#### 2.2.1. Internet addiction

The Revised Chen Internet Addiction Scale (CIAS-R) was used to measure Internet addiction. CIAS-R was compiled by Ko et al. (2005), based on the DSM-IV diagnostic criteria for various addictions, clinical case observations, and interview results. Including tolerance, withdrawal symptoms, time management, compulsive Internet access and interpersonal and health, there are 26 items in total. The scale divided into two subscales: Core Symptoms of Internet Addiction and Related Problems of Internet Addiction. The symptoms of Internet addiction include Internet addiction tolerance, compulsive Internet use, and Internet implicit withdrawal reaction. The problems related to Internet addiction include time management, interpersonal, and health issues. CIAS-R is scored using a 4-point Likert scale (1 = extremely inconsistent to 4 = very consistent), with higher total scores indicating higher Internet addiction tendency. Referring to the demarcation criteria of Ko et al. (2005) and other scholars, a scale score of 64 or above was defined as Internet addiction (Ko et al., 2005). The internal consistency coefficient of the entire scale was 0.93, and those of the core symptoms and related problems subscales were 0.90 and 0.88, respectively, showing good overall reliability and validity (Lin et al., 2011).

#### 2.2.2. Depression

The Self-Rating Depression Scale (SDS; Zung et al., 1965) was used to measure depression. The internal consistency coefficient was 0.84, Pearson correlation coefficient was 0.778, and Spearman rank correlation coefficient was 0.783 for this measure. It contains 20 items and is scored on a 4-point scale where 1 = no or little time, 2 = sometimes, 3 = most of the time, and 4 = most or all of the time. Among the 20 items, 10 items are reverse-scored. The total score is calculated by adding the scores for the 20 items. The standard score is obtained by multiplying the total score by 1.25, and an index <50 indicated no depression; 50–59 indicated mild depression; 60–69 indicated moderate to severe depression; and ≥ 70 indicated severe depression. In China, an SDS standard score ≥ 50 is regarded as having depressive symptoms.

#### 2.2.3. Anxiety

The Self-rating Anxiety Scale (Zung, 1971) was used to measure anxiety. The 20-item scale had a Cronbach’s α of 0.767, Spearman Brown split half reliability coefficient of 0.724, and Guttman split half reliability coefficient of 0.720. Overall reliability was acceptable. The scoring method is similar to that of Zung et al. (1965) SDS Scale; five of the 20 items are reverse-scored. The standard score was obtained by multiplying the total score by 1.25, with higher scores indicating higher anxiety levels. According to the standard score, anxiety level was classified as follows: < 50 points, no anxiety; 50–59 points, mild anxiety; 60–70 points, moderate anxiety; and ≥ 70 points, severe anxiety.

### 2.3. Statistical analysis

SPSS 22.0 was used for data entry and management, and SPSS 22.0 and Amos 22.0 were used for data entry and management and statistical analysis. Statistical significance was set at *P*<0.05. Analyses included descriptive statistics, *t*-tests, *F*-tests, Pearson’s correlation analyses, and cross-lagged analyses.

## 3. Results

### 3.1. Characteristics of participants

Baseline descriptive statistics of demographics, depression, anxiety and internet addiction are shown in Table 1. The baseline participants included 90 men and 153 women, and 243 in total (19.65 ± 0.83 years).

**Table 1:** Baseline descriptive statistics of demographics, depression, anxiety, and internet addiction.

		*N*	*M* (SD)
Sex	Male	90	
	Female	153	
habitation	City	83	
	Countryside	160	
Only child	Yes	73	
	No	170	
Age			19.74 (0.94)
Internet addiction			57.82 (12.12)
Depression			50.08 (9.78)
Anxiety			43.51 (9.71)

### 3.2. Analysis of sex differences by variable

The average scores of male and female students at the three time periods and the t-test results of independent samples of depression, anxiety, and Internet addiction among male and female college students are shown in Table 2. The results showed that depression (T1, T2, and T3) and anxiety (T1, T2, and T3) differed significantly by sex, whereas Internet addiction (T1, T2, and T3) did not.

**Table 2:** Analysis of sex differences in depression, anxiety, and Internet addiction.

	Males	Females	*t*	*P*
Internet addiction (T1)	58.41 (13.62)	57.48 (11.19)	0.55	0.58
Internet addiction (T2)	60.12 (9.62)	59.46 (7.66)	0.59	0.56
Internet addiction (T3)	56.41 (12.54)	54.16 (11.46)	1.43	0.16
Depression (T1)	52.86 (9.72)	48.44 (9.46)	3.48	0.00
Depression (T2)	53.04 (10.11)	48.14 (9.07)	3.90	0.00
Depression (T3)	52.01 (9.30)	48.26 (9.36)	3.03	0.00
Anxiety (T1)	45.87 (10.25)	42.13 (9.12)	2.95	0.00
Anxiety (T2)	46.34 (10.87)	41.29 (9.08)	3.71	0.00
Anxiety (T3)	50.91 (9.44)	47.02 (9.20)	3.15	0.00

T1, time 1; T2, time 2; T3, time 3.

### 3.3. Correlation analysis between variables

The results showed an effect of group (Table 3). In the overall group, depression, anxiety, and Internet addiction were positively correlated, among which T1 anxiety, T1 depression, T2 anxiety, and T2 depression had the highest correlation coefficients. Among women, a positive correlation between depression, anxiety, and Internet addiction were found at all three time points, and the correlation coefficients between T2 Internet addiction and T1 Internet addiction, T1 anxiety, and T1 depression were higher. Among men, depression, anxiety, and Internet addiction were positively correlated at all time points, and the correlations between T1 anxiety and T1 depression, T2 anxiety, and T2 depression were higher than that among women.

**Table 3:** Correlation analysis between depression, anxiety, and Internet addiction in the general, male, and female groups.

		1	2	3	4	5	6	7	8	9
Total	Internet addiction (T1)	1								
	Internet addiction (T2)	0.55**	1							
	Internet addiction (T3)	0.51**	0.34**	1						
	Depression (T1)	0.37**	0.27**	0.29**	1					
	Depression (T2)	0.34**	0.40**	0.27**	0.54**	1				
	Depression (T3)	0.31**	0.34**	0.43**	0.52**	0.52**	1			
	Anxiety (T1)	0.39**	0.27**	0.27**	0.70**	0.51**	0.50**	1		
	Anxiety (T2)	0.32**	0.43**	0.34**	0.49**	0.72**	0.51**	0.57**	1	
	Anxiety (T3)	0.24**	0.27**	0.42**	0.43**	0.52**	0.59**	0.54**	0.58**	1
Males	Internet addiction (T1)	1								
	Internet addiction (T2)	0.49**	1							
	Internet addiction (T3)	0.41**	0.21	1						
	Depression (T1)	0.36**	0.25*	0.16	1					
	Depression (T2)	0.39**	0.48**	0.23*	0.43**	1				
	Depression (T3)	0.26*	0.36**	0.42**	0.44**	0.39**	1			
	Anxiety (T1)	0.43**	0.28**	0.15	0.69**	0.47**	0.44**	1		
	Anxiety (T2)	0.31**	0.46**	0.27**	0.43**	0.72**	0.47**	0.45**	1	
	Anxiety (T3)	0.28**	0.31**	0.38**	0.32**	0.44**	0.55**	0.39**	0.50**	1
Females	Internet addiction (T1)	1								
	Internet addiction (T2)	0.61**	1							
	Internet addiction (T3)	0.59**	0.45**	1						
	Depression (T1)	0.39**	0.28**	0.36**	1					
	Depression (T2)	0.30**	0.35**	0.27**	0.58**	1				
	Depression (T3)	0.34**	0.33**	0.43**	0.53**	0.57**	1			
	Anxiety (T1)	0.36**	0.26**	0.33**	0.69**	0.50**	0.51**	1		
	Anxiety (T2)	0.33**	0.41**	0.37**	0.48**	0.70**	0.51**	0.63**	1	
	Anxiety (T3)	0.20*	0.24**	0.43**	0.46**	0.53**	0.58**	0.61**	0.61**	1

T1, time 1; T2, time 2; T3, time 3. *p < 0.05. **p < 0.01.

### 3.4. Variance analysis test of variables at different time points

Table 4 shows the results of the variance analysis of variables at different time points. The results showed an effect of group; the mean values of Internet addiction (*F* = 20.96, *p* < 0.001) and anxiety (*F* = 51.29, *p* < 0.001) at the three time points were significantly different; however, the mean value of depression at the three time points was not significantly different (*F* = 0.27, *p* = 0.77).

**Table 4:** ANOVA test of variables at different time points.

Variable	Time	M ± SD	*F*	*p*	Multiple mean comparison
Internet addiction	T1	57.82 ± 0.78	20.96	<0.001	T2>T1>T3
	T2	59.70 ± 0.54			
	T3	55.00 ± 0.76			
Depression	T1	50.08 ± 0.63	0.27	0.77	
	T2	49.95 ± 0.62			
	T3	49.65 ± 0.61			
Anxiety	T1	43.52 ± 9.71	51.29	<0.001	T3>T1>T2
	T2	43.16 ± 10.06			
	T3	48.46 ± 9.46			

T1, time 1; T2, time 2; T3, time 3.

### 3.5. Cross-lagged analyses

**Figure 1:**
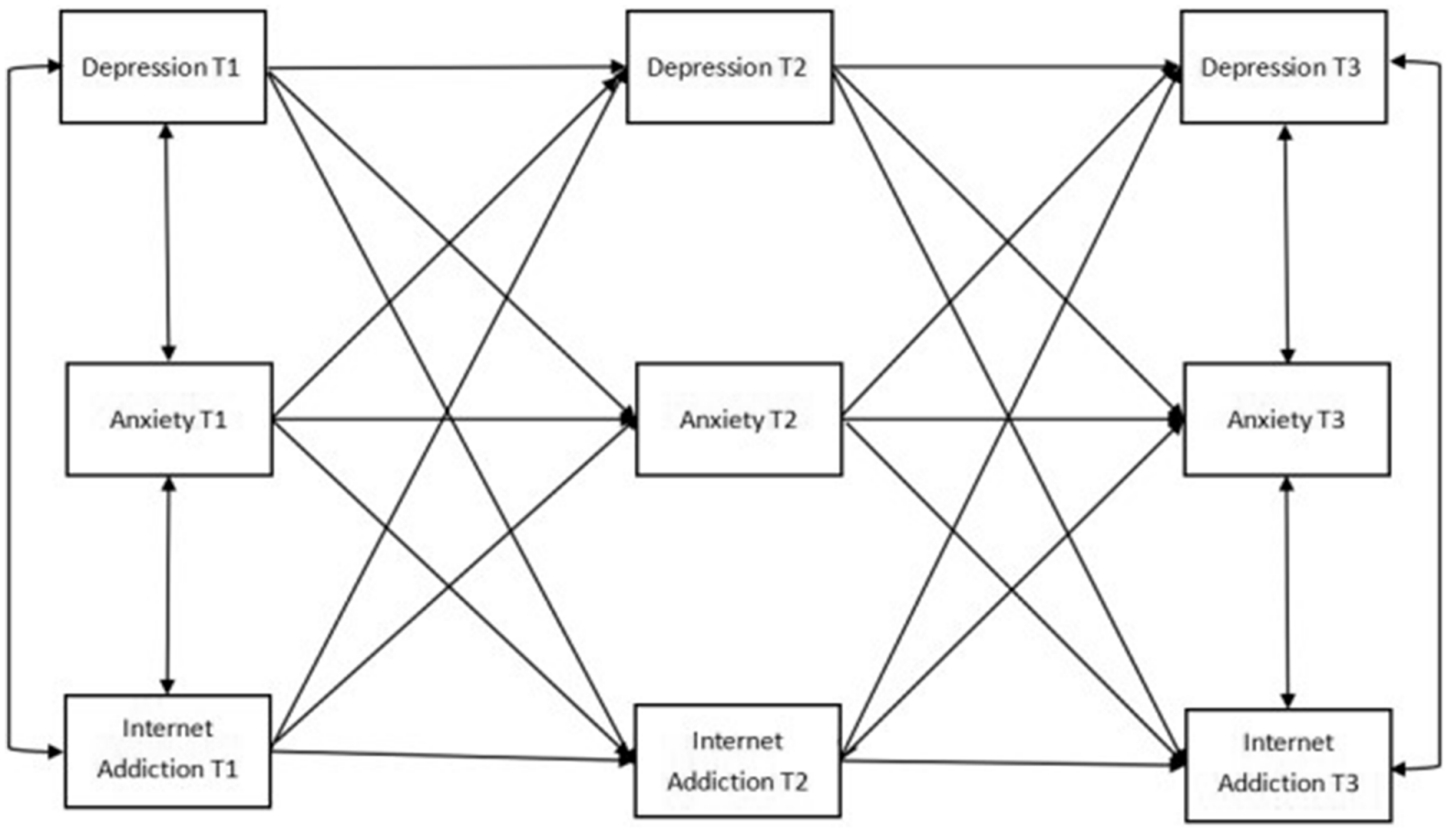
Cross-lagged theoretical model of depression, anxiety, and Internet addiction.

**Figure 2:**
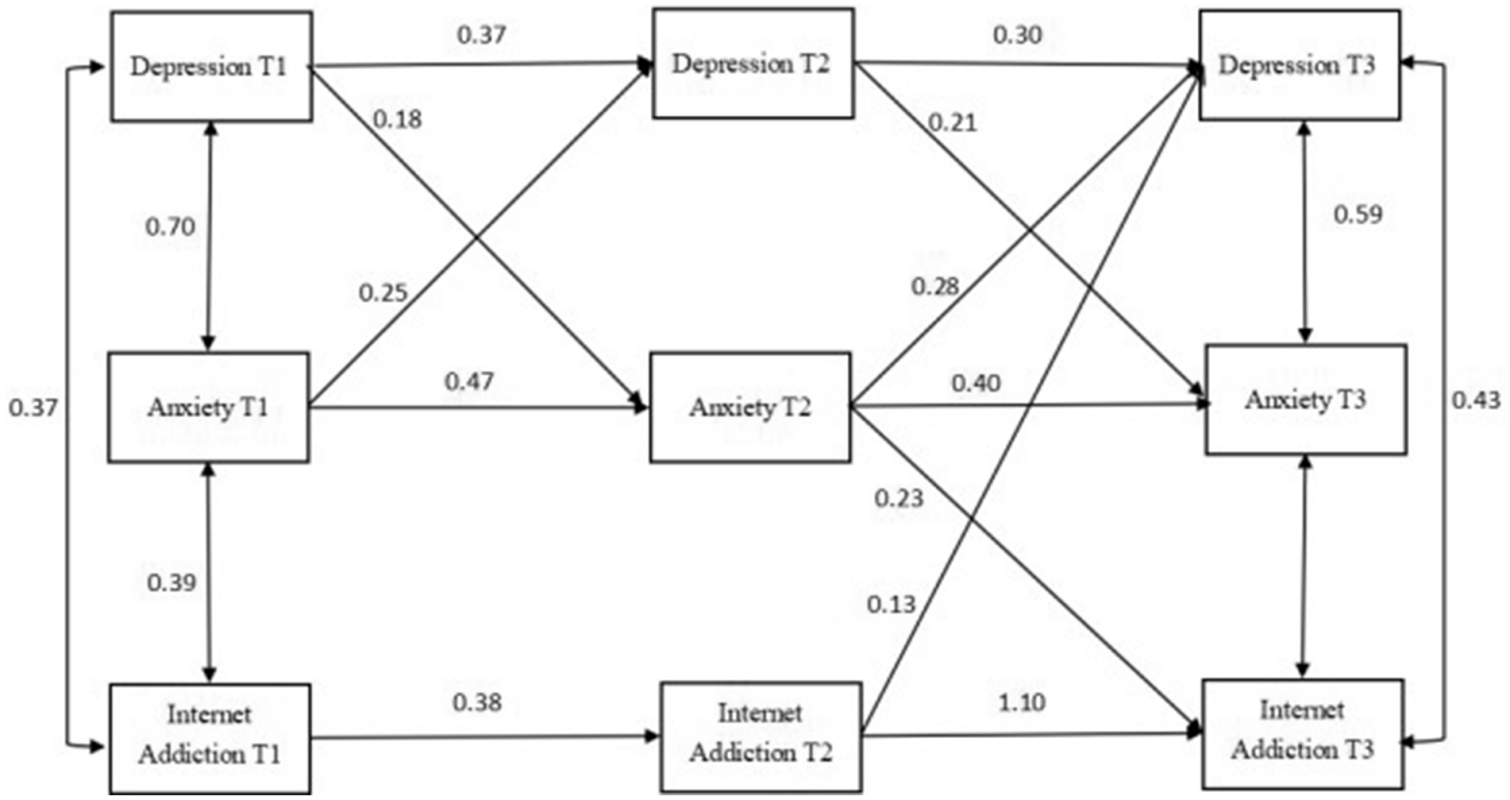
Cross-lagged analysis of depression, anxiety, and Internet addiction. T1 is the baseline data and T2 and T3 are follow-up data. The solid line indicates statistical significance.

**Figure 3:**
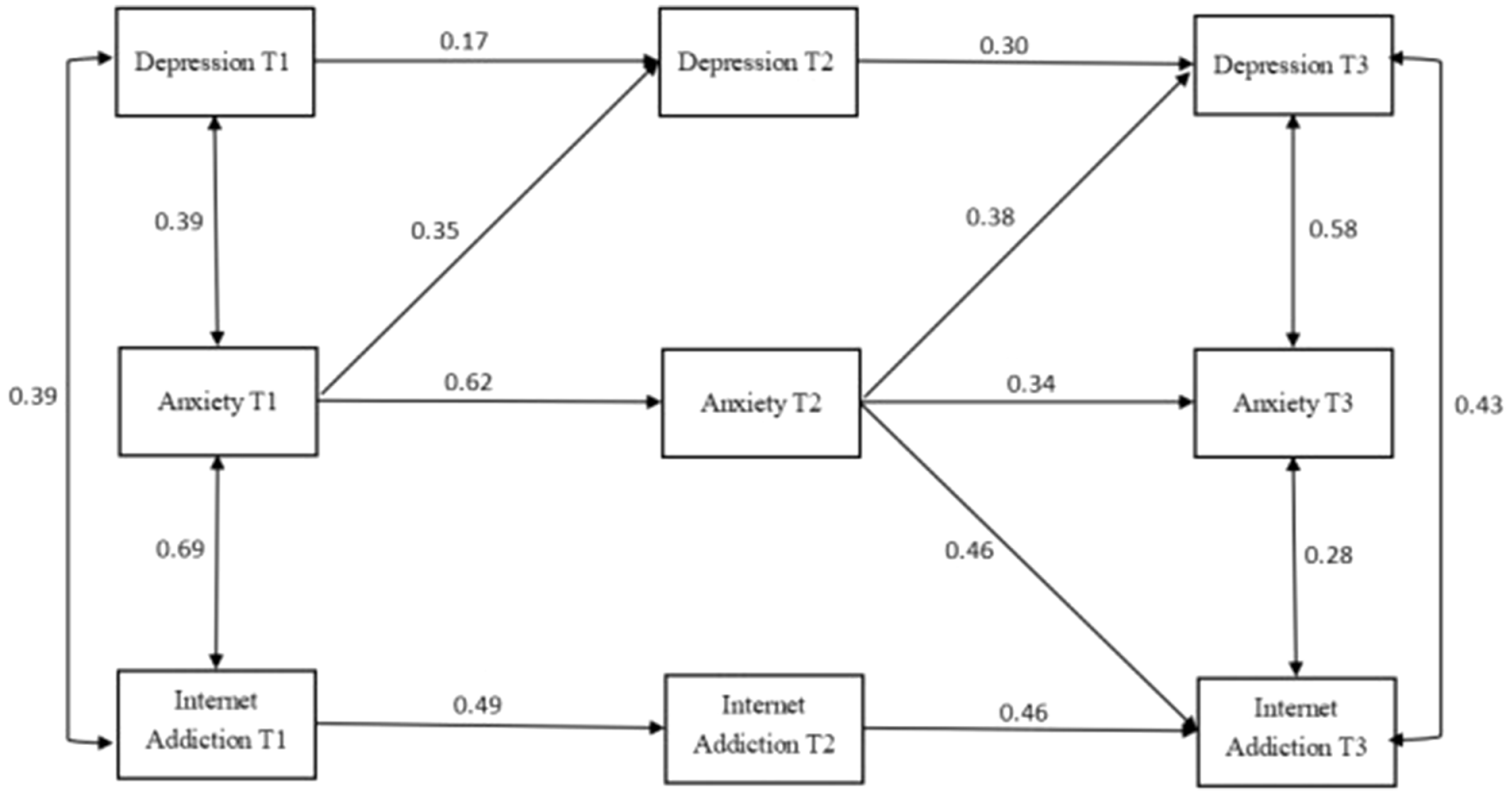
Cross-lagged analysis of depression, anxiety, and Internet addiction among men. T1 is the baseline data and T2 and T3 are follow-up data. The solid line indicates statistical significance.

**Figure 4:**
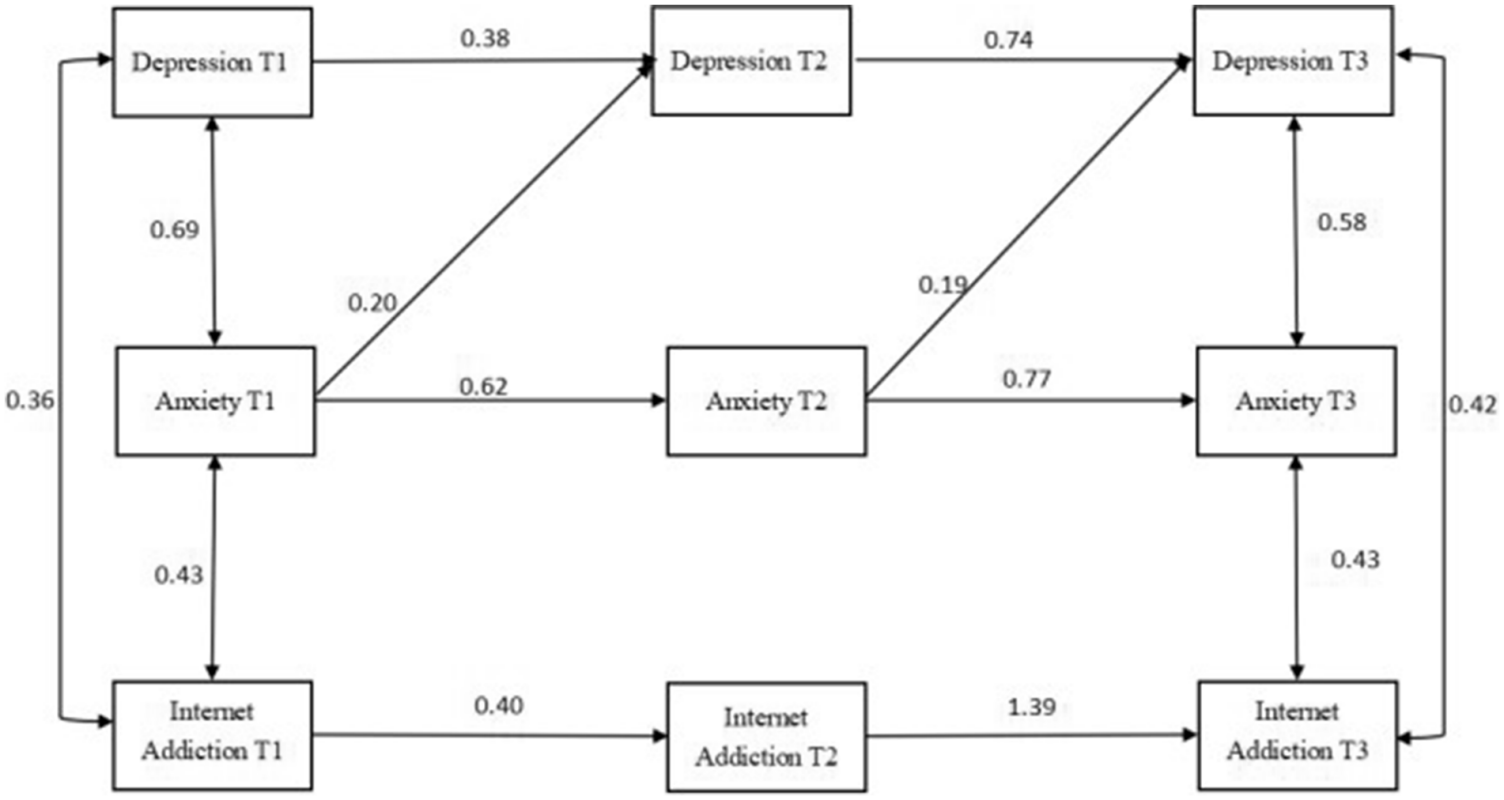
Cross-lagged analysis of depression, anxiety, and Internet addiction among women. T1 is the baseline data and T2 and T3 are follow-up data. The solid line indicates statistical significance.

The theoretical model of cross-lagged analysis was first constructed in this study in accordance with the previous literature (Figure 1; Krossbakken et al., 2018; Teng et al., 2021). After running each group (i.e., total, male, and female) combined with the theoretical composition and hint of correction coefficient, the final model diagram with good fit was obtained (Figures 2–4).

#### 3.5.1. Cross-lagged analysis of depression, anxiety, and internet addiction in the overall group

The cross-lagged model was used to test the relationship between depression, anxiety, and Internet addiction, measured at each time point. The model fit indices were good: CMIN = 37.692, CMIN/DF = 2.899, GFI = 0.969, AGFI = 0.891, CFI = 0.975, RMSEA = 0.089. As shown in Figure 1, T1 depression could positively predict T2 anxiety; T1 anxiety was a positive predictor of T2 depression; T1 Internet addiction could not predict T2 depression and T2 anxiety; T2 depression could positively predict T3 anxiety and T3 Internet addiction; T2 anxiety was a predictor of T3 depression and T3 Internet addiction; and T2 Internet addiction was a predictor of T1 depression but could not predict T3 anxiety.

#### 3.5.2. Cross-lagged analysis of depression, anxiety, and internet addiction among men

The cross-lagged model was used to test the relationship between depression, anxiety, and Internet addiction among men, measured at three time points. The model fit indices were good: CMIN = 21.530, CMIN/DF = 1.435, GFI = 0.952, AGFI = 0.855, CFI = 0.978, and RMSEA = 0.070. As shown in Figure 2, T1 anxiety can positively predict T2 depression but cannot predict T2 Internet addiction; T1 Internet addiction could not predict T2 depression or T2 anxiety; T1 depression could not predict T2 anxiety or T2 Internet addiction; T2 anxiety was a positive predictor of T3 depression and T3 Internet addiction; T2 depression could not predict T3 anxiety or T3 Internet addiction; and T3 Internet addiction could not predict T3 depression or T3 Internet addiction.

#### 3.5.3. Cross-lagged analysis of depression, anxiety, and internet addiction among women

The cross-lagged model was used to test the relationship between depression, anxiety, and Internet addiction among women, measured at three time points. The model fit indices were good: CMIN = 20.680, CMIN/DF = 1.477, GFI = 0.972, AGFI = 0.908, CFI = 0.990, RMSEA = 0.056. As shown in Figure 4, T1 anxiety could positively predict T2 depression but not T2 Internet addiction; T1 Internet addiction could not predict T2 depression or T2 anxiety; T1 depression could not predict T2 anxiety or T2 Internet addiction; T2 anxiety was a positive predictor of T3 depression but could not predict T3 Internet addiction; T2 depression could not predict T3 anxiety or T3 Internet addiction; and T3 Internet addiction could not predict T3 depression or T3 Internet addiction.

## 4. Discussion

In recent years, Internet addiction among college students has been widely studied. Researchers have proposed a close relationship between Internet addiction and college students’ emotional problems. The descriptive statistics show the high detection rate of internet addiction among college students. This is related to that they are the main group of network users (Nagaur, 2020). Because most students live in the dormitory of the school, they are far away from their families, relatives and friends, so they spend more time on online entertainment and communication (Baturay and Toker, 2019). Moreover, they also need to complete most learning tasks through the network.

The correlation analysis of this study show that depression, anxiety, and Internet addiction are positively correlated, which is consistent with previous studies (Mak et al., 2018; Geng et al., 2021). These results indicate an internal relationship among the three, providing further support for their longitudinal relationship.

Gender differences can be found in many addictive behaviors and their related factors, including Internet addiction. The present study show that men are more prone to Internet addiction than women. This difference might be owing to the different ways men and women use the Internet: men may focus more on online games, whereas women may focus more on online shopping, novel reading, and interpersonal communication (Liang et al., 2016). Both men and women can experience Internet addiction, but there are differences in their manner and purpose of Internet use as well as the content of their subsequent addictions, and the way men surf the Internet contains more addictive content. In previous research by this research group, it was also found that more of men’ online behaviors may be concentrated in online games, and more of women’s online behaviors may be concentrated in online shopping, novel reading, and interpersonal communication (detailed data can be obtained from the author).

This study used a half a year longitudinal design to gain insights into the role of gender in the association between depression, anxiety and Internet addiction. We found that the causal relationship between Internet addiction and anxiety varies by sex. Moreover, the causal relationship between Internet addiction and depression also varies by sex.

For male college students, Internet addiction can significantly predict the occurrence of later depression, but depression does not significantly predict Internet addiction. These results support the Internet addiction leads to depression in males. Some evidence suggests the gender differences in depression that women are more likely to have depressive symptoms than men (Lin et al., 2021). Several theorists have suggested that gendered processes of socialization affect how some boys and men express depression (Swetlitz.2021). In contrast, in this study, for males, Internet addiction is a risk factor for depression (Diotaiuti et al., 2022a). According to the previous studies, excessive Internet use has a negative influence on real-world social interactions, including reducing the scale of social circle (Mak et al., 2018). Some studies have found that Internet addicted men have less social contact in the offline world, which usually leads to depression (Paudel et al., 2021). Moreover, anxiety significantly predicts Internet addiction among male college students, this is consistent with previous follow-up research results (Tsai et al., 2020). This suggests that anxiety is a risk factor for Internet addiction among male college students (Shan et al., 2021). This is consistent with our research hypothesis.

For female college students, Internet addiction does not significantly predict the occurrence of later anxiety, and anxiety does not significantly predict the occurrence of later Internet addiction also. This is inconsistent with previous conclusions (Kim et al., 2021). Moreover, there was no causal relationship between depression and Internet addiction, contrary to the results of a previous study (Yi and Li, 2021). Depression was not a significant predictor of Internet addiction, and Internet addiction was not a predictor of depression. This is inconsistent with our research hypothesis.

The cross-lagged analysis indicated that anxiety was a predictor of depression among both men and women, but depression did not significantly predict anxiety; there was no two-way predictive relationship between anxiety and depression. According to the cross-lagged analysis, college students’ depression could predict anxiety in the overall group, indicating that individuals with high depression levels are more likely to face anxiety problems. Additionally, college students’ anxiety could predict depression, indicating that individuals with high anxiety levels are also more likely to experience subsequent depression. Depression and anxiety have a two-way predictive relationship. The results for the overall group show that anxiety can predict Internet addiction, which in turn could significantly predict depression, aligning with existing research (Hsueh et al., 2019; Gao et al., 2021). When college students experience great psychological pressure and develop anxiety about their studies and lives, they will often use the Internet to escape pain, vent about their anxiety, and obtain temporary psychological satisfaction (Javaeed et al., 2019). Internet use for short periods of time may temporarily relieve individuals’ anxiety, but long-term dependence on the Internet can cause more feelings of social disconnection and aggravate depression when facing real life (Swetlitz, 2021).

Although this study has some achievements, some limitations must still be addressed. First, the sample was from a single university in Southwest China, which limits the universality of the results. Second, the span of the three time points was short; future research should use more time points over a longer period to gain detailed understanding of the interaction of variables over time. Third, this study only investigated the degree of Internet addiction and did not analyze the differences in Internet addiction behaviors, such as gaming and shopping. In the future, we will conduct a thorough analysis of different Internet addiction behaviors.

This study achieved some expected results through system tracking and cross-lagged analysis. The results indicate that to develop Internet addiction interventions, attention should be paid to anxious individuals’ frequency and duration of Internet use. Corresponding policies should be formulated to guide the use of diversified ways to alleviate anxiety. The results also suggest that prevention and intervention strategies for Internet addiction should be designed for different sexes. Special attention should be paid to men, and targeted strategies and methods should be provided to alleviate anxiety when intervening in Internet addiction.

## 5. Conclusion

A cross-lagged study was used to analyze the relationships among depression, anxiety, and Internet addiction at three time points. The results showed that anxiety was a predictor of Internet addiction, and that Internet addiction could significantly predict depression. The results also showed that the relationship between Internet addiction and anxiety varies by sex. Male anxiety had a significant predictive effect on Internet addiction.

## Data availability statement

The raw data supporting the conclusions of this article will be made available by the authors, without undue reservation.

## Ethics statement

Written informed consent was obtained from the individual(s) for the publication of any potentially identifiable images or data included in this article.

## Author contributions

XX, HC, and ZC designed the study and drafted the manuscript. XX and HC analyzed the data and discussed the results. XX and ZC revised the manuscript. All authors contributed to the article and approved the submitted version.

## Conflict of interest

The authors declare that the research was conducted in the absence of any commercial or financial relationships that could be construed as a potential conflict of interest.

## Publisher’s note

All claims expressed in this article are solely those of the authors and do not necessarily represent those of their affiliated organizations, or those of the publisher, the editors and the reviewers. Any product that may be evaluated in this article, or claim that may be made by its manufacturer, is not guaranteed or endorsed by the publisher.
